# Resistance to EGFR inhibitors: Molecular determinants and the enigma of head and neck cancer

**DOI:** 10.18632/oncotarget.407

**Published:** 2011-12-31

**Authors:** Luc G.T. Morris, Timothy A. Chan

**Affiliations:** Human Oncology and Pathogenesis Program, Head and Neck Service, Department of Surgery, Memorial Sloan-Kettering Cancer Center, NY, USA; Human Oncology and Pathogenesis Program, Department of Radiation Oncology, Memorial Sloan-Kettering Cancer Center, NY, USA

The epidermal growth factor receptor (EGFR or ERBB1), is aberrantly activated in many human malignancies, including colorectal cancer, non-small cell lung cancer, and head and neck squamous cell carcinoma (HNSCC). In these diseases, overexpression correlates with poor survival. Inhibitors of EGFR signaling, both monoclonal antibodies (cetuximab, panitumumab) and tyrosine kinase inhibitors (TKIs; erlotinib, gefitinib), demonstrate activity in these cancers, but responses are heterogeneous. Resistance to EGFR-inhibiting agents continues to pose a substantial obstacle to their effective use. Many tumors do not initially respond, indicative of intrinsic resistance; of those responding, most eventually progress, demonstrating acquired resistance. In the treatment of HNSCC, cetuximab, in combination with radiation therapy, achieves substantial rates of response[1]. However, the response rate to single-agent cetuximab is only 10-15%; to erlotinib, 5%[2, 3]. Molecular predictors of response to EGFR inhibition in HNSCC remain poorly defined.

Several determinants of response to EGFR inhibitors have been characterized in lung and colorectal cancer. In lung cancer, molecular determinants were presaged by the realization that a specific clinically-definedsubpopulation (Asian, female, never-smokers, adenocarcinomas) responded best to TKIs. Subsequently, EGFR mutations associated with TKI sensitivity (exon 19 and L858R) or resistance (T790M) were identified[4]. In colorectal cancer, KRAS mutations were found to be associated with cetuximab resistance[5]. In both lung and colorectal cancers, EGFR copy number predicts response to cetuximab somewhat, but the predictive value is not high. Although not yet in clinical use, preclinical data has also implicatedresistance mechanisms such as VEGF signaling, AKT/mTOR pathway activation, and “oncogenic shift” to other receptor tyrosine kinases such as ERBB2, ERBB3, MET or IGF-1R, via overexpression or increased ligand availability[6].

In contrast, our understanding of mechanisms underpinning resistance to EGFR-targeted therapy is comparatively poor in HNSCC. Molecular determinants are not well defined. The most predictive factor for cetuximab sensitivity in HNSCC is a clinical finding – the development of a skin rash during treatment[1]. EGFR copy number is not predictive of response. Activating EGFR mutations are very rare, as are KRAS and BRAF mutations. Unlike in some other cancers such as GBM, the EGFRvIII variant does not predict response. Some promising insights have been reported recently, however. Preclinical data have demonstrated that increased expression of the ligand heparin-binding EGF-like growth factor (HB-EGF) occurs during the development of resistance in HNSCC cell lines, and that plasma HB-EGF levels are elevated in recurrent tumors[7]. There is also evidence that head and neck tumors can evade EGFR inhibition by undergoing epithelial-to-mesenchymal transition, thereby losing EGFR dependency.

Recently, frequent deletion of the *PTPRS* gene, encoding protein tyrosine phosphatase receptor S, was described in HNSCC[8]. A comprehensive genome-wide analysis of copy number alteration in HNSCC identified recurrent, intragenic microdeletions at the *PTPRS* gene locus in 26% of tumors. The focal nature of these deletions argues that *PTPRS* is the target of copy number alteration at chromosome 19p13. These deletions result in loss of protein expression of PTPRS, a membrane-bound phosphatase that dephosphorylates EGFR. Depletion of PTPRS leads to increased levels of phosphorylated EGFRand increasedEGFR signaling. Interestingly, loss of PTPRS, and consequently increased EGFR phosphorylation, renderscancer cells significantly more resistant to EGFR inhibitors. In fact, in normally TKI-sensitive HNSCC and lung cancer cells, knockdown of PTPRS is sufficient to induce erlotinib resistance. PTPRS seems to play a similar role modulating cetuximab resistance in HNSCC cells. Interestingly, clinical outcome is also dramatically influenced by PTPRS status. Patients with lung adenocarcinomas harboring activating EGFR mutations *and*low PTPRSexpression have significantly poorer survival thansimilar patients with normal PTPRS-expressing tumors.

Together, these recent data demonstrate that a frequent genetic event in HNSCC, *PTPRS* loss, is able to help drive EGFR pathway activation, and modulate sensitivity to EGFR inhibitors. With additional clinical investigation, these findings may open the door to the possibility of *PTPRS* status serving as a biomarker for drug resistance, analogous to EGFR or KRAS resistance mutations in lung and colorectal cancer. This might aid in triaging patients to EGFR inhibitors or conventional chemotherapy. TKI trials, limited to sensitive EGFR mutations in lung cancer, have achieved impressive response rates of 50-70%. Ultimately, overcoming these novel mechanisms of resistance in HNSCC –loss of *PTPRS* or persistent levels of EGFR activity – will prove instrumental in enhancing tumor response to these promising agents.
